# Development of a Structured Interview to Explore Interpersonal Schema of Older Adults Living Alone Based on Autobiographical Memory

**DOI:** 10.3390/ijerph18052316

**Published:** 2021-02-26

**Authors:** Yunna Kwan, Sungwon Choi, Tae Rim Eom, Tae Hui Kim

**Affiliations:** 1 Department of Psychiatry, Wonju Severance Christian Hospital, Wonju 26426, Korea; kwbl8902@gmail.com; 2 Department of Psychology, Duksung Women’s University, Seoul 01369, Korea; karatt92@duksung.ac.kr; 3 Health Insurance Research Institute, National Health Insurance Service, Wonju 26464, Korea; Taerim0923@naver.com; 4 Department of Psychiatry, Yonsei University Wonju College of Medicine, Wonju 26426, Korea

**Keywords:** interpersonal relationship, interpersonal schema, older adults, structured interview

## Abstract

With a growing public interest in the social health of older adults, studies focusing on social networks and interpersonal relationships of older adults are needed. The present study was conducted to develop a structured interview to evaluate the interpersonal schema based on Self-Defining Memory of older adults. First, the word cues that the older adults often report on interpersonal events were confirmed. Next, the indices and scoring rules were prepared, including Relationship frequency (RF), Conflict frequency (CF), Dominance mean (Dm), and Warmth mean (Wm). Healthy older adults living alone (mean age = 71.81, SD = 3.95) were interviewed. Finally, the correlation between each index and Short form of Korean Inventory of Interpersonal Problems Circumplex Scales (KIIP-SC) was analyzed for criterion validity. The inter-rater reliability was substantial (Kappa = 0.61~0.66). Based on the analysis of criterion validity, the indices of CF, Dm, and Wm indices showed an appropriate level of criterion validity. This study developed a structural interview based on a novel system of reporting autobiographical memory and established indices with appropriate validity to evaluate interpersonal relationships. The interview is expected to identify the characteristics of interpersonal relationships of the older adults and contribute to the establishment of the older adults’ community accordingly.

## 1. Introduction

The paradigm for health is shifting along with the increment of human life expectancy through advances in medicine [1]. For the health policy makers of today, it will be a key task to pay attention to the social aspects of health and to avoid factors that hinder personal and social health. In this regard, one of the emerging issues is that the number of older adults living alone is increasing exponentially with the aging society [2]. It is thought to be related to new social problems including deaths due to loneliness and social isolation of the older adults living alone [3]. Accordingly, the policy to protect against social isolation by developing an older adults’ community has become a global trend in recent years [4,5,6]. Most of these older adults’ communities are based on geographic proximity [4,5]. However, the natural development and maintenance of interpersonal relationships are not only shaped by simple geographic proximity, but are also affected by various interpersonal factors as individual characteristics, schema, and attitudes toward interpersonal relationships, and shared lifestyles and interests [7,8,9]. Hence, it is important to identify factors that affect the interpersonal relationships for a successful older adults’ community.

**Figure 1:**
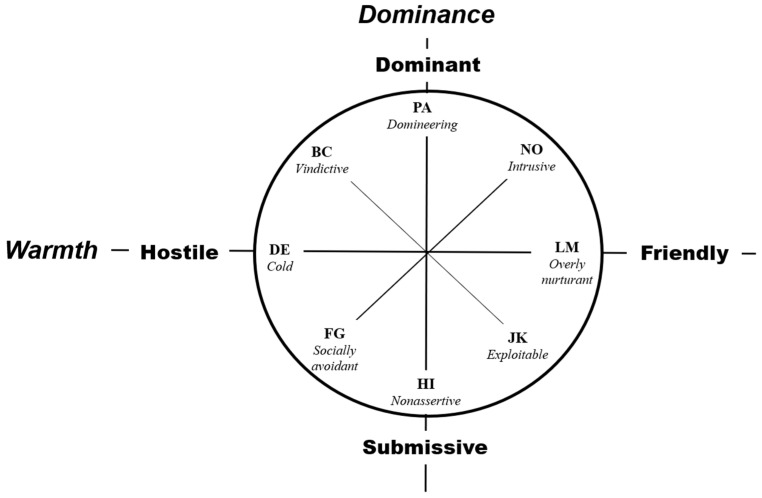
Interpersonal Circumplex Model [10,11]. Note: The interpersonal circumplex (IPC) model classified interpersonal schema and behaviors into eight octants under orthogonal two axes (Dominance and Warmth). Each octant was described as Domineering (PA), Vindictive (BC), Cold (DE), Socially avoidant (FG), Nonassertive (HI), Exploitable (JK), Overly Nurturant (LM), and Intrusive (NO) characteristics.

The interpersonal schema is considered as a decisive factor that fundamentally affects both the initiation and maintenance of interpersonal relationships among many other factors influencing interpersonal relationships [7]. The interpersonal circumplex model (IPC) describes the interpersonal behaviors based on two orthogonal fundamental domains: Dominance (or agency, control) and Warmth (or communion, affiliation) [10,11]. The model hypothesizes that interpersonal schema and behavior represent the interaction between domination/submission and friendliness/hostility. The individual differences in interpersonal relationships are classified into eight sectors (or octants) under these two axes. The octants with high relevance are adjacent to each other, are located at an angle of 90° if they are unrelated, and are located at an angle of 180° if they exhibit opposite characteristics (Figure 1). Accordingly, the more right means the more intimate and pro-social the attitude, and the more left, the more hostile and colder the attitude in the Figure 1. The higher means the more dominant and leading attitude in interpersonal relationships, and the downward the more submissive and non-assertive attitude toward others likewise. When the model is simply divided into quadrants, the upper right represents an intimate and proactive attitude with others, the lower right represents an intimate and obedient attitude, the upper left represents a cold, hostile, and assertive attitude towards others, and the lower left represents hostile, but non-assertive, and submissive [10,11].

The advantage of the IPC model is that it is not only directly related to the conceptualization and evaluation of personality disorders [12], but is also consistent with personality classification according to the five-factor theory, enabling a broad interpretation of the interpersonal behaviors of healthy and clinical population [13,14]. Researchers tried to place traits derived from different personality theories on the IPC topology. For instance, the extraversion of the five-factor theory is not exactly an interpersonal trait, but still it is located on the upper right section of the IPC model, related to a relatively friendly but intrusive attitude toward others [15,16]. On the other hand, agreeableness is related to the attitude of being pro-social and trusting others and is located on the lower right section of the IPC model [15,16]. Evidence also suggested that the IPC model is associated with a variety of pathological characteristics. Chronic depression, more severe forms of social anxiety, and Schizoid personality disorder are primarily associated to the lower left area of the IPC model [17,18,19]. Personality traits such as pathological narcissism, psychopathy, and Machiavellianism have also occupied their own positions on the IPC topology, respectively [20].

Considering that the importance of interpersonal relationships is constantly being emphasized in the mental health of older adults, literature focusing on interpersonal relationships of and evaluation tools specific to the older adults based on IPC is insufficient despite the high utility of the model. Moreover, various scales used to assess interpersonal schema based on IPC have been developed and used for clinical and research applications to date, but these scales are still limited by their self-reported features using paper and pencil [11,21]. In addition to the general disadvantages of self-reporting assessment tools, such as the impact of social desirability and problems caused by individuals not really understanding themselves well [22], the paper-delivered tests raise realistic concerns especially in the older adults, including the high illiteracy rate of the older adult population compared to younger adults, which may interfere with the response of the older adults to questions [23,24]. Further, the paper and pencil-based self-reported tests are associated with inherent limitations, as personalization is difficult because the item content is limited to general situations and interpersonal relationships [22]. An objective measure that has greater ecological valid than paper and pencil-based self-reported tests would be needed to evaluate the interpersonal schema in older adults.

Self-Defining Memory (SDM) is a part of autobiographical memory, but is a more specific, vivid, emotionally intense memory, suggesting that self-discovery enables self-understanding, and contributes to a sense of identity [25]. Exploring SDM would be helpful not only to increase the ecological validity based on individual experience, but also to utilize the strengths of the IPC model in the assessment of interpersonal schema, because it reflects the continuing interests and unresolved conflicts of individuals, and contains a core belief (schema) of oneself and the world [26]. In particular, SDM for interpersonal events is formed by experiences and behaviors in individual relationships to date, which can again influence future interpersonal behaviors [27,28]. It is considered as appropriate for the evaluation of interpersonal relationships and serves as an important predictor of individual behavior in future relationships. Many researchers have developed several types of interview and performance evaluation tools to assess autobiographical memories [29,30]. However, most of the measurements have been developed for the purpose of identifying disease-specific impairment of autobiographical memory, with limited validity for evaluation of the extent and degree of impairment such as the accuracy and specificity of memory, and impossible to evaluate core beliefs or relationship schema quantitatively [29,31].

We aimed to develop a structured interview for evaluating the Interpersonal schema based on Autobiographical Memory (IAM) of older adults living alone as part of a study of expanding social network for the older adults living alone. In particular, we tried to develop an interview suitable for the evaluation of interpersonal schemas, while not being too broad in the content of the reported SDM. To this end, we standardized the word cues to those of life events involving interpersonal relationships that elders living alone often report. In addition, in order to systematically measure the interpersonal schema according to the two dimensions of Dominance and Warmth based on the IPC, we developed scoring rules according to the typical interpersonal schema and behaviors of each axis based on previous studies [21].

Accordingly, we aimed to identify the coordinates of the reported SDMs on the IPC topology, and to test whether the indexes developed in this study agree with the Dominance and Warmth dimensions by examining the concurrent validity with well-established tests developed based on the IPC model. More specific, the Dominance index score was hypothesized to be related to the indicators corresponding to the top and bottom of the IPC model, and the Warmth index score was thought to be related to the indicators corresponding to the left and right side. We also wanted to exploratively identify the relationship between the frequency of SDM reports related to interpersonal relationships and conflicts and the IPC model.

## 2. Materials and Methods

### 2.1. Development of the Structured Interview

#### Literature Review and Initial Development

Cue item generation was conducted via a systematic review of assessment tools based on the Self Memory System (SMS) [32], a theoretical model of autobiographical memory (AM) that is supported by evidence. Previous studies have evaluated semantic memories and schema of AM using word cues, mainly presented as emotional words or events that people commonly experience in their lives. In this study, items were created using a list of life events as cues in order to facilitate the retrieval of self-referential memories by the older adults [33,34,35]. Berntsen and Rubin [33] provided a list of 35 cultural and universally experienced events in a lifetime. In this study, ‘other’ items were added to consider the specificity of Korean older adults. A structured interview was constructed based on a total of 36 derived cues. Questions and prompts for each question were designed to enable the Korean older adults to understand based on a review of a geriatric psychiatry specialist and two licensed clinical psychologists, who also are authors of this research. Every question and prompt was visually presented to increase the attention and memory of cue words [36,37].

### 2.2. The Pilot Study

The purpose of the pilot study was to identify the content categories of AM mainly reported by the older adults living alone in Korea, determine the word cue appropriate for assessing the interpersonal schema, and determine the interview structure. Most of the previous studies on AM and SDM of healthy people used general cue words because older people were not the only focus of research, suggesting limitations that cues were not only overly broad, but also unsuitable for the older adults. Therefore, we tried to standardize the word cues appropriate for the older adults living alone.

First, convenience sampling of the older adults living alone in Wonju City and aged 65 to 80 years based on the census data of the Ministry of Health and Welfare (MoHW), confirming participation by telephone. In order to adjust for the role of cognitive decline in the understanding of tasks and recall of autobiographical memory, individuals whose mini-mental state examination-dementia screening (MMSE-DS) [38,39] had scores of −1.5 sd or below compared to age and educational norm, or those who were diagnosed with major neurocognitive disorders, were excluded. The total number of participants was 30 (Females = 21, mean age = 71.73 years, SD = 3.67). Based on a review of the 36 cards, participants were instructed to choose and freely talk about 3 to 5 most memorable events in their lives within 2 h without any restriction. Interviewers were three students who were enrolled in their master’s course, majoring in clinical psychology, and the interviews were conducted under the supervision of a licensed clinical psychologist. The entire interview was recorded with the participants’ consent. In the analysis of the pilot study, 12 cues with a reported frequency of more than 20% were selected (Table 1), and all 12 cues were related to interpersonal relationships. Finally, the interview structure of IAM was established through agreement between one geriatric psychiatry specialist and two licensed clinical psychologists after reviewing the entire recording protocol.

### 2.3. Final Interview Protocol

In the final version of IAM, the categories for autobiographical memory were modified to a total of 10. As a result of the pilot study, ‘First job’ and ‘Earning money first time’ were merged because the participants showed similar reports under these two categories, and ‘Marriage’ and ‘Divorce’ were also merged because these categories were reported similarly by the spouses. From among 10 thematic category cards, three important events in the participants’ life were selected and reported.

Next, the following scoring rules were prepared for scoring and coding individual interpersonal schema.

Relationship frequency (RF): As several researchers have revealed, the frequency of reporting SDMs for interpersonal relationships was thought to be important in identifying the self-image or adaptive behavior of individuals related to interpersonal relationships [40,41]. When the content of SDM included interpersonal relationships in each category, a score of 1 was coded. The total RF score was in the range of 0–3.

Conflict frequency (CF): This index was also developed with reference to previous studies. CF reflects an individual’s interpersonal self-image. It is known that individuals tend to find meaning from interpersonal conflicts, and represent behaviors based on these lessons in the next interpersonal relationship [26,41]. A score of 1 was coded when there was at least one expressed reference to the fight, disagreement, or disappointment caused by a conflict of desire or goal between at least two characters (not necessarily including participants), and the total score was in the range of 0–3.

Finally, each reported SDM was scored according to the typical behavior index of the Dominance and Warmth axes [21]. The Dominance and Warmth scores for each question ranged between −1.0 and 1.0. Higher scores on the Dominance axis indicated SDM for dominant behavior in interpersonal situations, and lower scores indicated SDM for submissive behavior. Higher scores on the Warmth axis suggested that SDM for intimate and friendly behavior in interpersonal situations, and lower scores indicated SDM for cold and indifferent or even hostile behavior. The average score of the interpersonal schema reported under each cue was used as indexes, so that the average location of the individual interpersonal schema was identified on the quadrant consisting of two dimensions.

Dominance index mean value (Dm): This index indicates location of the average value of the interpersonal SDMs reported by the individual on the Dominance axis. Thus, it tells where the individual’s interpersonal schema is posited on the line of domination–obedience.

Warmth index mean value (Wm): This index indicates the location of the average value of the interpersonal SDMs reported by the individual on the Warmth axis. In other words, Wm represents where the individual’s interpersonal schema is located on the line of friendliness–hostility.

### 2.4. Participants

Similar to the pilot study, the study involved older adults living alone in Wonju city and aged between 65 and 80 years based on the census data of the MoHW for the older adults living alone. First, among the individuals who did not participate in the pilot study, 150% of the participants were allocated based on the dropout rate via stratified random sampling according to the residential area (urban vs. rural) and gender. Only individuals who agreed to participate in the study through telephone contact were recruited. In order not to be disturbed by cognitive decline in the understanding of tasks and recall of autobiographical memory, individuals whose mini-mental state examination-dementia screening (MMSE-DS) [38,39] had scores of −1.5 SD or below compared to age and educational norm, or who were diagnosed with major neurocognitive disorder, were excluded. In addition, since depressed mood was known to affect the recollection of autobiographical memories in older adults [42], the depressed older adults who scored 10 or more in the geriatric depression scale-short form (SGDS) [43] were also excluded. Participant demographic information is presented in Table 2.

This study was conducted with the approval of the institutional ethics committee of Yonsei University Wonju Severance Christian Hospital (protocol #CR318026).

### 2.5. Additional Outcome Measures

MMSE-DS [38,39]: It is a 30-point questionnaire used to screen dementia. It consists of brief items used to evaluate time and place orientation, memory registration and recall, attention, naming, command execution, repetition, visuospatial ability, understanding, and judgment.

SGDS [43]: It is a shortened form of a 30-item older adults depression scale developed by Yesavage [44] to measure older adults’ depression. The sensitivity and specificity of sGDS for depression screening has been reported to be comparable to that of conventional GDS. The total score ranged between 0 and 15. 

Agreeableness subscale of Big Five Inventory (BFI-A) [45,46]: BFI was developed to evaluate five personality traits (extraversion, agreeableness, openness, conscientiousness, and neuroticism) according to the Big Five personality theory. Among them, the agreeableness subscale is used in this study, which evaluates personality traits related to pro-social behaviors such as trust, altruism, kindness, and affection for others. Since BFI-A measures an interpersonal behavior pattern that is relatively pervasive, it was conducted to investigate the concurrent validity of IAM, developed to identify interpersonal schema and behavior.

Short form of Korean Inventory of Interpersonal Problems Circumplex scales (KIIP-SC) [47]: KIIP-SC is a shortened version of the Inventory of Interpersonal Problems Circumplex scales (IIP-C) developed by Alden, Wiggins, and Pincus [48] based on the IPC model. IIP-C has been used to comprehensively evaluate interpersonal issues and identify the most important interpersonal issues. The KIIP-SC is a self-report test of 80 questions and consists of 8 subscales: Domineering subscale (PA) measures issues related to controlling and manipulating others; Vindictive subscale (BC) measures challenges related to excessive interest in one’s well-being; Cold subscale (DE) evaluates problems related to emotional experience and expression; Socially avoidant subscale (FG) measures difficulties related to non-social tendencies and shyness; Nonassertive subscale (HI) measures lack of self-confidence, assertiveness, and self-esteem; Exploitable subscale (JK) is used to assess behaviors related to persuasion and exploitation without maintaining independence; Overly Nurturant subscale (LM) measures issues caused by excessive sensitivity and altruism; and Intrusive subscale (NO) assesses behaviors related to excessive involvement with others. KIIP-SC is composed of a total of 40 items based on 5 questions that best reflect each of the 8 factors, and the internal consistency (Cronbach’s α) ranged from 0.61 to 0.89 among college students and adults. KIIP-SC was conducted to examine the concurrent validity of IAM, which aimed to identify the interpersonal schema based on IPC model.

### 2.6. Procedure

Since the measure developed in this study is a structured interview, the interviewers were trained in the interview and scoring methods in advance, and obtained sufficient practice before conducting the IAM. The interviewers were composed of college graduates working as life managers of older adults living alone and were familiar with interviewing the older adults.

This study was based on 1:1 interviews at a place convenient for the study participants (home or outpatient clinic at a university hospital located in Wonju). Written explanations and oral explanations were provided so that the participants fully understood and provided informed consent before participation in the interviews. Structured interviews took place about 30 min after MMSE-DS and SGDS, followed by BFI-A and KIIP-SC. Every protocol used in the analysis was written verbatim. The interpersonal schema was scored by two licensed clinical psychologists independently. One was the author of this study, the other was well trained for this interview. Both had sufficient clinical experience for the geriatric population.

### 2.7. Statistical Analysis

The data were analyzed using SPSS 25.0 (IBM Corp., Armonk, NY, USA). First, descriptive statistics were analyzed to identify participants’ demographic information. Next, Cohen’s Kappa coefficients of indexes in the structured interview scored by each rater were tested for inter-rater reliability. The interpretation of the inter-rater reliability was as follows [43]: >0.00 = poor; 0.00 to 0.20 = slight; 0.20 to 0.40 = fair; 0.40 to 0.60 = moderate; 0.60 to 0.80 = substantial; 0.80 to 1.0 = almost perfect. If the raters’ scores were inconsistent, the average was calculated and adjusted for the next analysis. Then the item-total correlation was calculated by Pearson’s correlation analysis. Finally, Pearson’s correlation analysis between different subscales of KIIP-SC, BFI-A, and sub-indexes of IAM (RF, CF, Dm, and Wm) was performed to examine the concurrent validity, which is a type of criterion validity and could be tested through correlation analysis with measures considered similar. Three participants who did not report any SDM related to interpersonal relationships were excluded from concurrent validity analysis.

## 3. Results

Table 2 shows demographic information of participants, with a total of 62 women and 37 men completing the interview.

### 3.1. Inter-Rater Agreement of IAM

Table 3 shows Cohen’s Kappa coefficients for reliability of indexes scored independently by the two raters. All of the RF, CF, D, and W indexes showed substantial agreement (RF Kappa = 0.66, *p* < 0.001; CF Kappa = 0.61, *p* < 0.001; D Kappa = 0.62, *p* < 0.001; W Kappa = 0.62, *p* < 0.001).

### 3.2. Item-Total Correlation

To examine the item-total correlation of Dm and Wm indexes, we conducted Pearson’s correlation analysis (Table 4). Each item of indexes showed a significant correlation with the index scores.

### 3.3. Criterion Validity

Next, Pearson’s correlation analysis was performed to examine the correlation between sub-indexes of IAM (Table 5). The RF showed a significant positive correlation only with CF (r = 0.37, *p* < 0.001). Meanwhile, CF showed a significant correlation with Dm and Wm (Dm r = 0.21, *p* < 0.05; Wm r = −0.68, *p* < 0.001).

To examine the validity of the criterion, the correlation between the Sub-indexes of IAM, Agreeableness subscale of BFI, and subscales of KIIP-SC was calculated (Table 5). First, the RF did not show any significant correlation with other measures. CF showed a significant negative correlation with the BFI-A and positive correlation with DE, FG, and NO subscales of KIIP-SC (BFI-A r = −0.30, *p* < 0. 05; DE r = 0.24, *p* < 0.05; FG r = 0.25, *p* < 0.05; NO r = 0.42, *p* < 0.001). Next, Dm showed a significant negative correlation with BFI-A and positive correlation with the NO subscale of KIIP-SC (BFI-A r = −0.23, *p* < 0.05; NO r = 0.21, *p* < 0.05), and Wm showed a significant negative correlation with the DE and FG subscales of KIIP-SC (DE r = −0.24, *p* < 0.05; FG r = −0.25, *p* < 0.05).

## 4. Discussion

In this study, a structured interview was developed to evaluate the Interpersonal schema based on Autobiographical Memory (IAM) of older adults living alone as part of a study of expanding social network for the older adults living alone, and the reliability and validity of the ratings were examined. It was organized based on a search of autobiographical memories, overcoming the limitations of the self-report scale, and provided personalized evaluation using events that are important to individuals. In addition, a word list that frequently reported self-defining memories related to interpersonal relationships was provided for the older adults living alone in Korea, so that the interview evaluated their interpersonal schemas more efficiently.

First, to confirm the reliability of the interview, inter-rater reliability of IAM and item-total correlation of Dm and Wm indexes were identified. The substantial reliability of all indexes suggested that the agreement between the raters of indexes for the interpersonal schema based on the interview was sufficiently reliable. In addition, the item-total correlation of the two indexes was also significantly high. Therefore, the scoring method provided by the structured interview tool developed in this study represents a stable method for the evaluation of interpersonal schema based on the exploration of personal self-defining memories.

Next, in order to examine the validity of the structured interview, the correlation between the sub-indexes of the interview was determined, and the relationship with the KIIP-SC sub-scale was analyzed. The relationship frequency (RF), a level of reporting on the content of interpersonal relation in important self-defining memories, did not show any significant correlation with other indexes, except the conflict frequency (CF) reporting interpersonal conflicts. Since there was no significant association with any of the subscales of KIIP-SC, which collects responses to difficulties in interpersonal relationships, the RF was considered insufficient to identify problematic or maladaptive interpersonal schemas. However, the reported interpersonal SDM’s frequency is suggested to be important in identifying individuals’ self-image related to interpersonal relationships [40,41]. It is thought that the predictive validity of this index could be examined through further research, rather than immediate discarding. RF can also be used as a way to evaluate the validity of the entire interview. In other words, indexes obtained through interviews with an RF value of 0 should be considered unreliable.

CF showed a significant negative correlation with the mean value of the Warmth index (Wm), which measures the point on the scale of behaviors from intimate and compassionate to cold and indifferent in interpersonal behavior. It showed a significant positive correlation with the mean value of the Dominance index (Dm), which measures the point on the continuous line from the dominant behavior to the submissive behavior in interpersonal relationships. In other words, individuals who report more memories of conflicts related to interpersonal relationships, whether or not they are involved in the conflict, are expected to be more likely to exhibit relatively cold behavior and were indifferent in interpersonal relationships as well as behaviors controlling or dominating others.

Correlation between the subscales of IAM, BFI-A, and KIIP-SC was confirmed to analyze the concurrent validity. The CF score showed a significant negative correlation with BFI-A and positive correlation with the DE, FG, and NO subscales of KIIP-SC, suggesting this index might be closely related with relatively maladaptive interpersonal schemas and behaviors. The older adults who recalled conflict situations more frequently among other memories are expected to be relatively hostile or cynical to others since CF is related to the DE and FG subscales of KIIP-SC, which are located on the hostile side of the Warmth axis of the IPC model. On the other hand, it seems difficult for them to show appropriate behavior even when they want to be friendly to others. Older adults who represent high CF likely to have difficulty in expressing pro-social behaviors such as intimacy, altruism, and empathy in interpersonal situations, while also being excessively intrusive with others’ behavior.

This study revealed that frequent memories of conflict could not serve as lessons for satisfactory interpersonal relationships, at least in the older adults, and suggested that CF is a useful index associated with dysfunctional interpersonal schemas. It is known that SDM for interpersonal conflict reflects the interpersonal self-image that individuals have for themselves, and also serves as a manual for coping in relationships with others, whether it is positive or negative [26,41]. For instance, researches examining the memory of patients with Borderline personality disorder (BPD) have discovered that patients frequently recall SDM for failed trust by family members or romantic partners [49], and that they recall negative social information much better [50,51]. These characteristics of memories seem to eventually serve as a manual for violent and unstable interpersonal behaviors of BPD patients. Further research is needed on whether CF can discriminate personality disorders, still it was revealed that this index has a high sensitivity to a vindictive and hostile schema to others (Table A1).

The Dm score, measured in autobiographical memory selected as important to oneself, showed a negative correlation with BFI-A and significant positive correlation with the NO subscale of KIIP-SC, which measures issues related to excessive involvement with others. Thus, individuals exhibiting more proactive and dominant behavior in interpersonal relationships based on autobiographical memories more frequently experienced problems involving interference with and engaging in actions with others, which was consistent with the location of NO subscale on a high level of Dominance axis in the IPC model [47,52]. Moreover, it was found that those who scored high in the Dm index exhibited a low level of agreeableness. Agreeableness is a personality trait that represents social adaptability and communal attributes toward others, and includes qualities such as altruism, affection, trust, consideration, and humility. Although agreeableness and dominance were derived from independent theoretical concepts of personality and interpersonal schema, respectively, several researchers have explored their relevance [15,53]. Agreeableness is known as located in relatively friendly and submissive direction (i.e., lower right section in Figure 1) on the IPC model [15], which is consistent with the negative correlation between Dm index and Agreeableness revealed as results of this study. 

The results suggest that older adults who scored high in the Dm index could behave dysfunctional as being overly interfering with others rather than trusting others or acting altruistically, even though they have a compassionate interest in others and want to behave pro-socially, considering the NO subscale has both high Dominance and Warmth values in the IPC model. This kind of behavior might be problematic when they want reciprocal social relationships. Previous studies have shown that highly dominant people prefer relationships in a harsher, hierarchical way, and frequently use coercion, intimidation, and power to gain high social status [54,55]. However, these behaviors are likely to cause different outcomes than their intentions. From our findings, it can be speculated that interventions related to social skills and pro-social behavior will be useful for older adults with high scores on the Dm index.

The Wm score showed a significant negative correlation with the DE and FG, rep-resenting challenges related to socially distant and cold, or excessively shy and evasive attitudes, respectively. This negative correlation suggests that the Wm index is highly valid for the assessment of behavior in the Warmth axis since the DE and FG is a parameter located at the bottom of the Warmth axis in the IIP model (i.e., left side of Figure 1). The results also suggest that older adults who score low on this index may be aloof, avoidant, and even hostile toward others. This kind of attitude might be an important factor to cause older adults’ maladjustment in communities. In fact, Machiavellianism, one of the Dark Triad personalities that can seriously harm social relationships, sits in a submissive–hostile position in the IPC model [20]. Individuals with high levels of Machiavellianism easily manipulate others for their own interests, and basically have cynical and distrustful interpersonal schemas [20,56]. In addition, social workers might need to carefully monitor the mental health of older adults who score high in Wm in community. A lot of studies have focused on the vulnerability in mental health of individuals with submissive–hostile interpersonal schemas. Although clinical level depression was excluded in this study, researchers have shown that submissive–hostility is common in chronically depressed patients [17], and is associated with more severe social anxiety [19].

Contrary to our hypothesis, however, the Dm score did not show a significant correlation with the PA subscale of KIIP-SC, which is used to measure challenges related to controlling and manipulating others and located at the highest level on the Dominance axis. Moreover, no negative correlation was observed between the Dm score and the HI, the nonassertive subscale of KIIP-SC, located at the bottom of the Dominance axis. the Wm score did not show significant correlation with the LM subscale of KIIP-SC, which measures issues related to excessive responsibility for the needs of others, and placed at the highest sides on the Warmth axis of the IPC model. The results can be explained as due to the primary focus of KIIP-SC are on problematic behaviors and schemas reviled in interpersonal relationships. It is necessary to understand the terms of interaction with the individual environment whether or not an individual’s main behavioral patterns in interpersonal relationships caused problems [57,58]. Thus, the older adults living alone who increasingly reported the dominant and leading behaviors based on a high Dm score showed intimacy with others, but likely manifested frequent issues related to invasive and excessive interference behavior, rather than challenges related to intentional control and manipulation of others [44,47]. Likewise, individuals who show high Wm scores could behave pro-social and friendly at an appropriate level, so that might not be related to significant interpersonal problems.

Lastly, the indexes developed in this study did not show high sensitivity to maladaptive interpersonal schema, but specificity was found to be very high (Table A1). It could serve as an advantage of IAM, considering that this interview was not a diagnostic test tool, but aimed to promote healthy interpersonal relationships by identifying the interpersonal schema of older adults who do not report specific problems with interpersonal relationships.

The study limitations are as follows. First, the study participants were limited to the older adults living alone in small and medium-sized towns, and therefore do not represent a large city or rural area. It is possible that the characteristics of the residential area were reflected in the interpersonal behaviors and schemas reported by participants. This limitation was addressed by recruiting participants according to the demographic characteristics of the older adults living alone in Wonju city, and determining the schematic of SDM and interpersonal relations according to actual interpersonal relationship patterns of the older adults living alone. Second, although well-established tests were used to examine the concurrent validity of the structured interview developed in this study, still further studies are needed to determine whether the interpersonal schematic indexes can predict the real-world interpersonal behavior of the older adults living alone. Finally, the interviewers and the raters of the interview were separated, and the raters only read the written verbatim for scoring, since this study was conducted as a part of a research project providing emotional support for the older adults living alone. As a result, it is possible that the reliability of the evaluation is somewhat questionable because nonverbal communication clues such as facial expressions, intonation, and gestures were not considered [59]. The inter-rater reliability may be enhanced by providing structured manuals and formal training to enable implementation and scoring, and direct evaluation of the interviewer’s method.

## 5. Conclusions

This study developed a structured interview to evaluate interpersonal schemas based on autobiographical memory, and analyzed its reliability and validity. Until now, most of the measures of interpersonal relationship schemes relied on self-reports, which are limited by the effects of social desirability, insufficient ecological validity, and the inability to be used by the illiterate. We addressed this limitation using a brief interview method, and a more personalized evaluation strategy based on autobiographical memories. In addition, this study is meaningful in that it uses standardized cue words appropriate for the older adults living alone in Korea to systematically evaluate interpersonal relationships, and developed and validated indicators according to the IPC model, which is actively used in clinical and research fields. Based on the interviews developed in this study, it is expected that the characteristics of interpersonal relationships among the older adults living alone can be understood and used for social and public goals such as establishing older adults communities as well as in research applications.

## Figures and Tables

**Table 1:** Word Cues.

No.	Category	%	No.	Category	%
1	Serious illness	36.67	7	Marriage	23.33
2	Having grandchildren	36.67	8	Divorce	23.33
3	Death of parents	33.33	9	Baptism	20.00
4	Spousal bereavement	26.67	10	Socializing with peers	20.00
5	First job	26.67	11	Earning money for the first time	20.00
6	Brothers/sisters	23.33	12	Procreation	20.00

Note: Ordered by frequency of appearance.

**Table 2:** Demographic characteristics (*n* = 100).

Variables	Mean (Range) or %	SD
Age (year)	71.50 (65–79)	3.72
Gender (female; %)	63	
Education (year)	7.31 (0–20)	4.15
Period of living alone (year)	18.23 (1–47)	10.77
MMSE-DS	26.73 (20–30)	2.24
SGDS	3.09 (0–9)	2.73

Note. MMSE-DS = mini-mental status examination-dementia screening; SGDS = Geriatric depression scale-short-form.

**Table 3:** Inter-rater agreement of the Interpersonal schema based on autobiographical memory (IAM) sub-indexes.

Indexes	Kappa	*p*
RF	0.66	0.000
CF	0.61	0.000
Dominance index	0.62	0.000
Warmth index	0.62	0.000

Note. RF = Relationship Frequency; CF = Conflict Frequency.

**Table 4:** Correlation between the Interpersonal schema sub-indexes and subscales of Short form of Korean Inventory of Interpersonal Problems Circumplex scales (KIIP-SC).

Indexes	D1	D2	D3	W1	W2	W3
Dm	0.65 *	0.61 *	0.45 *	-	-	-
Wm	-	-	-	0.74 *	0.70 *	0.75 *

Note. Dm = Dominance index mean value; Wm = Warmth index mean value; * = *p* < 0.05.

**Table 5:** Correlation between the Interpersonal schema sub-indexes, Agreeableness subscale of Big Five Inventory (BFI-A), and subscales of Short form of Korean Inventory of Interpersonal Problems Circumplex scales (KIIP-SC).

	1	2	3	4	5	6	7	8	9	10	11	12	13
1. RF	-												
2. CF	0.37 **	-											
3. Dm	−0.01	0.21 *	-										
4. Wm	−0.09	−0.68 **	0.03	-									
5. A	−0.07	−0.30 *	−0.23 *	0.19	-								
6. PA	0.19	0.35 **	0.11	−0.19	−0.62 **	-							
7. BC	−0.05	0.08	0.08	−0.05	−0.28 *	0.36 **	-						
8. DE	−0.07	0.24 *	−0.01	−0.21*	−0.14	0.18	0.65 **	-					
9. FG	0.04	0.25 *	−0.03	−0.25*	−0.31 *	0.34 **	0.49 **	0.70 **	-				
10. HI	0.03	0.20	0.06	−0.10	−0.31 *	0.19	0.57 **	0.71 **	0.55 **	-			
11. JK	0.02	0.13	0.04	−0.12	−0.15	0.35 **	0.38 **	0.45 **	0.48 **	0.51 **	-		
12. LM	0.07	0.13	0.01	−0.01	−0.47 **	0.28 *	0.36 **	0.36 **	0.45 **	0.40 **	0.75 **	-	
13. NO	0.11	0.42 **	0.21 *	−0.17	−0.39 **	0.55 **	0.21 *	0.21 *	0.41 **	0.18	0.50 **	0.50 **	-
Mean	2.56	0.90	−0.56	0.63	44.61	9.00	11.88	13.53	11.45	14.24	13.37	13.96	10.30
Sd	0.63	0.81	0.69	1.21	4.35	2.47	2.97	3.69	3.13	3.56	3.28	2.94	4.35

Note. RF = Relationship Frequency index; CF = Conflict Frequency index; Dm = Dominance index mean value; Wm = Warmth index mean value; A = Agreeableness subscale of BFI; PA = Domineering subscale of Short form of Korean Inventory of Interpersonal Problems Circumplex scales(KIIP-SC); BC = Vindictive subscale of KIIP-SC; DE = Cold subscale of KIIP-SC; FG = Socially avoidant subscale of KIIP-SC; HI = Nonassertive subscale of KIIP-SC; JK = Exploitable subscale of KIIP-SC; LM = Overly Nurturant subscale of KIIP-SC; NO = Intrusive subscales of KIIP-SC; * = *p* < 0.05; ** = *p* < 0.001.

## Data Availability

Not applicable.

## Appendix A

**Table A1:** Classification accuracy for maladaptive interpersonal schemas by IAM indexes.

Variables (%)	Indexes	Sensitivity	Specificity	Positive Likelihood Ratio
PA (15.2)	Dm	0.0	100.0	84.0
	Wm	0.0	100.0	84.0
	CF	0.0	100.0	84.0
	Dm + Wm + CF	0.0	100.0	84.0
BC (51.5)	Dm	64.6	43.5	54.3
	Wm	64.6	32.6	48.9
	CF	70.8	28.3	50.0
	Dm + Wm + CF	54.2	54.3	54.3
DE (47.4)	Dm	0.0	100.0	53.5
	Wm	48.8	75.5	63.0
	CF	39.5	73.5	57.6
	Dm + Wm + CF	48.8	73.5	62.0
FG (30.2)	Dm	0.0	100.0	69.2
	Wm	7.1	93.7	67.0
	CF	10.7	100.0	72.5
	Dm + Wm + CF	17.9	95.2	71.4
HI (40.6)	Dm	0.0	100.0	59.3
	Wm	0.0	100.0	59.3
	CF	27.0	94.4	67.0
	Dm + Wm + CF	35.1	85.2	64.8
JK (30.9)	Dm	0.0	100.0	69.6
	Wm	3.6	100.0	70.7
	CF	0.0	100.0	69.9
	Dm + Wm + CF	3.6	100.0	70.7
LM (40.4)	Dm	0.0	98.2	59.6
	Wm	37.8	86.0	67.0
	CF	27.0	89.5	64.9
	Dm + Wm + CF	32.4	86.0	64.9
NO (10.1)	Dm	0.0	100.0	89.4
	Wm	0.0	100.0	89.4
	CF	0.0	100.0	89.4
	Dm + Wm + CF	20.0	100.0	91.5

Note. Dm = Dominance index mean value; Wm = Warmth index mean value; CF = Conflict Frequency index; PA = Domineering subscale of Short form of Korean Inventory of Interpersonal Problems Circum-plex scales (KIIP-SC); BC = Vindictive subscale of KIIP-SC; DE = Cold subscale of KIIP-SC; FG = Socially avoidant subscale of KIIP-SC; HI = Nonassertive subscale of KIIP-SC; JK = Exploitable subscale of KIIP-SC; LM = Overly Nurturant subscale of KIIP-SC; NO = Intrusive subscales of KIIP-SC.

Sensitivity, specificity, and positive likelihood ratio by IAM indexes for maladaptive interpersonal schemas were calculated. Maladaptive interpersonal schemas were defined as T70 or greater (i.e., 2 standard deviations from the mean) on each subscale of KIIP-SC.
